# Assessment of psychometric properties, cross-cultural adaptation, and translation of the Turkish version of the ICU mobility scale

**DOI:** 10.3906/sag-2005-319

**Published:** 2021-06-28

**Authors:** İsmail ÖZSOY, Buse ÖZCAN KAHRAMAN, Turhan KAHRAMAN, Aylin TANRIVERDİ, Serap ACAR, Ebru ÖZPELİT, Bihter ŞENTÜRK, Bahri AKDENİZ, Sema SAVCI

**Affiliations:** 1 Department of Physiotherapy and Rehabilitation, Faculty of Health Sciences, Selçuk University, Konya Turkey; 2 Department of Cardiopulmonary Physiotherapy-Rehabilitation, School of Physical Therapy and Rehabilitation, Dokuz Eylül University, İzmir Turkey; 3 Department of Physiotherapy and Rehabilitation, Faculty of Health Sciences, İzmir Katip Çelebi University, İzmir Turkey; 4 Department of Cardiology, Faculty of Medicine, Dokuz Eylül University, İzmir Turkey

**Keywords:** ICU mobility scale, functional status, intensive care unit

## Abstract

**Background/aim:**

The aim of the study was to carry out the cultural adaptation and translation of the ICU mobility scale (IMS) into Turkish and research the psychometric properties.

**Materials and methods:**

This study was based on methodological design. The IMS was translated from English to the Turkish through a regularised translation process. Two physiotherapists assessed patients independently in the coronary intensive care unit. The measures such as construct validity, intra and interrater reliability, and internal consistency of the IMS Turkish version were assessed.

**Results:**

A total of 70 intensive care patients were included in the study. The intrarater and interrater reliability of the IMS was excellent. The weighted Kappa value was 0.92 (0.87–0.96) for the intrarater reliability, and 0.87 (0.80–0.93) for the interrater reliability. There were significant correlations between the IMS and functional status score for the intensive care unit (r = 0.83), Perme intensive care unit mobility score (r = 0.84), Katz activities of daily living (r = 0.73), handgrip strength (r = 0.62), knee extension strength (r = 0.46), and age (r = –0.44).

**Conclusion:**

This study suggests that the IMS Turkish version is a reliable and valid scale for assessing functional status and mobility level in ICU patients.

## 1. Introduction

Intensive care units (ICUs) are special units where close follow-up and treatment of patients are performed in the presence of a life-threatening critical illness [1]. Early mobilization has been established as an important, feasible, and safe method to reduce the incidence of ICU-acquired weakness, increase functional capacity, and reduce hospital and ICU stay [2]. Early mobilization is a step-by-step process from rolling to independently walking to improve recovery and outcomes [3]. Therefore, it is crucial to define the mobility levels of intensive care patients in a way that all healthcare professionals can use [4].

A systematic review demonstrated that a few instruments such as functional status score for the ICU (FSS-ICU), Perme ICU mobility Score, and ICU mobility scale (IMS) assess functional status in ICU [4]. However, the IMS is the initial instrument that has declared feasibility and interrater reliability for assessing the maximum mobility level of functional status in ICU survivors and the IMS is the most practical scale to evaluate functional status over other scales. [5]. 

The IMS is a quick and simple method to assess functional status in ICU survivors [5]. It was developed by a multidisciplinary team of researchers and clinicians [5]. Studies support validity, interrater reliability, and responsiveness of the IMS as a measure of functional status in ICU survivors [5,6]. The use of IMS in international studies has increased day by day and it has been translated into different languages [7,8]. However, there is no Turkish translation of the IMS. It is vital to know the level of early mobilization in the management of intensive care patients. To improve early mobilization of ICU survivors, there is a need for instruments in Turkish. Having an instrument to measure functional status will increase functional capacity, improve early deficiencies, and reduce hospital and ICU stay. Therefore, the study aimed to carry out the translation and cultural adaptation of the IMS into Turkish and research the psychometric properties.

## 2. Materials and methods

### 2.1. Study design and participants

This study was based on methodological design. The study was carried out in a 16-bed adult coronary ICU in Dokuz Eylül University Hospital. Patients who were 18 years old and over, awake, and had independent activities of daily living before ICU were included in the study. We excluded participants if they had baseline cognitive or physical impairment, or hemodynamic instability preventing mobility.

### 2.2. Translation and cross-cultural adaptation

According to a proposed guide, cross-cultural adaptation and translation of the IMS into the Turkish process were carried out [9]. The process mentioned below has been followed.

#### 2.2.1 Initial translation 

Two forward translations were made of the instrument from the English to the Turkish by two translators. One of the translators was bilingual (having Turkish as their mother tongue). While one of the translators was not aware of the study, the other translator was aware of study concepts.

#### 2.2.2. Synthesis of the translations

A synthesis of the two translators’ versions was conducted. A written report was prepared.

#### 2.2.3. Backward translation 

To be sure that the translation was expressing the same item content as the English version, back translation was performed by two independent translators. They were native English speakers and fluent in Turkish. 

#### 2.2.4. Expert committee 

The expert committee’s role was to consolidate all the versions of the questionnaire. While forming the expert committee, care was taken to bring together competent people from different fields. The expert committee consisted of 14 people in total. These were health professionals (two medical doctors and six physiotherapists), the translators (two forward and two back translators), one Turkish language teacher, and one primary school teacher. The prefinal version was prepared by this committee.

#### 2.2.5. Prefinal version

The last stage of the adaptation procedure was the pretest. The prefinal translated version was tested to recommend items for modification or deletion. Recent arrangements were made.

#### 2.2.6. Final version

The final version was developed by the expert committee. Some minor changes were conducted to make use more understandable. The final Turkish version of the IMS was created.

### 2.3. Data collection

Two physiotherapists (>5 years of experience in ICU) participating in the assessment process received training and instructions on how to assess the Turkish version of the IMS prior to initiation of the assessment process. All evaluations were carried out on the 3rd day during the ICU stay. Two physiotherapists evaluated the same patients separately for the interrater reliability of the IMS on the same day. The IMS were scored by two physiotherapists who were blinded to each other. The same patient was assessed by one of the physiotherapists after 1 h if the clinical condition of the patient is similar to the first evaluation for the intrarater reliability.

The demographic characteristics were recorded. Before the assessments, vital and hemodynamic signs were observed on ICU monitor.

#### 2.3.1. The ICU mobility scale

The IMS is a simple, quick, and ordinal scale. It consists of 11 different mobility levels in total. It ranges from passive mobilization (0 = lying in bed) to independent mobilization (10 = ambulation independently without help). As the IMS score increases, the level of mobility also increases [5].

#### 2.3.2 Functional status score for the intensive care unit 

The FSS-ICU has 5 mobility levels (from rolling to walking). Each section is scored between 0 and 7, and the total score is a maximum of 35. As the score increases, the patient’s mobility level also increases [10]. It has been demonstrated that Turkish version of the FSS-ICU for the intensive care unit instrument is a valid and reliable scale [11].

#### 2.3.3. Perme intensive care unit mobility score

The Perme ICU mobility score contains 7 categories and 15 items. It is scored from 0 to 32 and a high total score indicates better mobility level [12]. It has been shown that the Turkish version of the Perme ICU mobility score is a valid and reliable scale [13].

#### 2.3.4. Peripheral muscle strength

An electronic hand-held dynamometer (Lafayette Instrument Company, Lafayette, IN, USA) was used to measure knee extensor muscle strength [14]. The Jamar hand dynamometer (Patterson Medical, Warrenville, IL, USA) was used to assess the handgrip strength [15]. Measurements were taken three times and average values were recorded.

#### 2.3.5. Katz activities of daily living (the KATZ ADL)

The KATZ ADL consists of 6 items. Each item is scored as 0 or 1, and the total score range is from 0 to 6. As the Katz ADL score increases, the independence of activities of daily living also increases [16]. It has been shown that the Turkish version of the Katz ADL is a valid and reliable scale [17].

### 2.4. Sample size

The minimum required sample size was calculated as 70 patients in this study. There is no generally acceptable consensus in the literature to calculate the minimum required sample size for validation studies. It is usually recommended to have 2–20 subjects per item [8]. 

### 2.5. Statistical analysis

The intra- and interrater reliability was investigated using the weighted Kappa statistic which was qualitatively interpreted as excellent (>0.8), strong (0.7–0.8), and good (0.6–0.7) [18]. 

Thirteen predefined hypotheses were determined to evaluate the construct validity, including convergent and discriminant. For testing the convergent validity, the following hypotheses were constructed: significant and high correlations between the IMS and (1) the FSS-ICU, (2) Perme ICU mobility, (3) Katz ADL, (4) handgrip strength-right, (5) handgrip strength-left; significant and moderate correlations with (6) knee extension strength-right, (7) knee extension strength-left, and (8) age. For testing the divergent validity, noncorrelations were expected between the IMS and (9) body mass index, (10) respiratory rate, (11) heart rate, (12) systolic blood pressure, and (13) diastolic blood pressure. These hypotheses were based on our clinical observations also supported by the previous studies [6,11]. Spearman’s rank correlation coefficients (rs) were calculated since the IMS is an ordinal variable. The correlation coefficients were interpreted as low correlation rs < 0.30, moderate correlation rs = 0.30–0.59, and high correlation rs ≥ 0.60 [19]. Statistical significance was set at p < 0.05. Data were analysed using the SPSS v. 22.0 programme (IBM Corp., Armonk, NY, USA).

## 3. Results 

A total of 70 intensive care patients were included in the study. Most of the participants were male (60%). Table 1 presents the patients’ characteristics and outcomes.

**Table1:** Characteristics of participants (n = 70).

Variables	Mean ± SD
Age (years)	69.65 ± 10.73
Sex (male, %)	42 (60)
Body mass index (kg/m2)	26.65 ± 3.84
Admission diagnosis (%)	
Acute coronary syndrome	38 (54.3)
Heart failure	11 (15.7)
Transcatheter aortic valve replacement	11 (15.7)
Implantable cardiac defibrillator implantation	7 (10)
Tachycardia	3 (4.3)
Respiratory rate (breaths/min)	19.59 ± 3.88
Heart rate (beats/min)	81.23 ± 22.06
SBP (mmHg)	117.37 ± 22.40
DBP (mmHg)	67.68 ± 11.57
Handgrip strength-right (kg)	23.55 ± 10.46
Handgrip strength-left (kg)	22.80 ± 10.53
Knee extension strength-right (kg)	14.87 ± 4.63
Knee extension strength-left (kg)	14.18 ± 4.21
Perme ICU mobility (score)	21.32 ± 5.09
Katz activities of daily living (score)	3.74 ± 1.53
Functional status score for the ICU (score)	24.27 ± 8.04
ICU mobility scale (score)	7.81 ± 2.04

SBP: systolic blood pressure, DBP: diastolic blood pressure, ICU: intensive care unit

It was found that the inter- and intrarater reliability of the IMS was excellent. The weighted Kappa value was 0.92 (0.87–0.96) for the intrarater reliability, and 0.87 (0.80–0.93) for the interrater reliability (Table 2). 

**Table 2:** Intra- and interrater reliability of the ICU mobility scale.

	Intrarater reliability[Kappa (95% CI)]	Interrater reliability[Kappa (95% CI)]
ICU mobility scale	0.92 (0.87–0.96)	0.87 (0.80–0.93)

ICU, intensive care unit; ICC, intraclass correlation coefficient, CI: confidence interval.

Significant and high correlations were observed between the IMS and the FSS-ICU, Perme ICU mobility, Katz ADL, handgrip strength (right), handgrip strength (left) (rs ≥ 0.60, p < 0.05). Significant and moderate correlations were observed between the IMS and knee extension strength (right), knee extension strength (left), and age (rs = 0.30–0.59, p < 0.05). 

Nonsignificant correlations between the IMS and body mass index, respiratory rate, diastolic blood pressure, and systolic blood pressure (p > 0.05). The IMS and heart rate was significantly and moderately correlated (rs = –0.37, p = 0.002). Twelve out of 13 predefined hypotheses were confirmed (92%) indicating that the construct validity of the IMS was good. Table 3 shows the predetermined hypotheses and correlation coefficients of the IMS with other measurements.

**Table 3:** Predetermined hypotheses, correlation coefficients of the ICU mobility scale with other measurements.

Type of validity	Variable	Hypothesis	Result	Confirmed
Convergent	Functional status score for the ICU	Significant and high correlation	0.83 (<0.001*)	Yes
Convergent	Perme ICU mobility	Significant and high correlation	0.84 (<0.001*)	Yes
Convergent	Katz ADL	Significant and high correlation	0.73 (<0.001*)	Yes
Convergent	Handgrip strength (right)	Significant and high correlation	0.62 (<0.001*)	Yes
Convergent	Handgrip strength (left)	Significant and high correlation	0.62 (<0.001*)	Yes
Convergent	Knee extension strength (right)	Significant and moderate correlation	0.46 (<0.001*)	Yes
Convergent	Knee extension strength (left)	Significant and moderate correlation	0.46 (<0.001*)	Yes
Convergent	Age	Significant and moderate correlation	–0.44 (<0.001*)	Yes
Divergent	Body mass index	Nonsignificant correlation	0.05 (0.664)	Yes
Divergent	Respiratory rate	Nonsignificant correlation	–0.04 (0.755)	Yes
Divergent	Heart rate	Nonsignificant correlation	–0.37 (0.002*)	No
Divergent	Systolic blood pressure	Nonsignificant correlation	0.16 (0.187)	Yes
Divergent	Diastolic blood pressure	Nonsignificant correlation	0.02 (0.851)	Yes

*Statistically significant.

## 4. Discussion

The present study demonstrates the initial report of psychometric properties, cross-cultural adaptation, and translation of the IMS in Turkish language. The results of this study showed that the IMS Turkish version has excellent inter- and intrarater reliability, construct validity, and internal consistency.

The IMS is a simple and quick bedside instrument to measure mobility in critically ill patients. In rehabilitation studies in the ICU, mobility milestones (e.g., first-time standing or walking) are commonly used as intermediate, functional endpoints. However, the IMS is a feasible tool with a sensitive 11-point ordinal scale, ranging from lying/passive exercises in bed (score of 0) to independent ambulation (score of 10) [6]. Evidence to support the reliability and face and content validity of the IMS is reported [5]. According to a previous study investigating the validity and responsiveness of various instruments, the IMS had criterion validity, could predict discharge destination, and could detect change over time from awakening to ICU discharge [20]. Furthermore, the predictive validity of the IMS in relation to 90-day mortality is also reported (1). It takes less than 1 min to complete the IMS [5]. Hodgson et al. demonstrated that the IMS has high interrater reliability with a weighted Kappa (95% confidence interval) of 0.83 (0.76–0.90) among junior and senior physiotherapists in surgical/trauma/medical ICU survivors [5]. Kawaguchi et al. showed that the IMS has excellent interrater reliability [Cronbach’s alpha coefficient (95% confidence interval) of 0.99] during the evaluation of survivors in 1 surgical ICU (20 beds) and 2 clinical ICUs (10 beds) [7]. A recent Spanish validation study of the IMS also showed that the Kappa index demonstrated values excellent for the IMS (Kappa index higher than 0.95) [8]. The Turkish version of the IMS has showed excellent interrater reliability in our study. Additionally, to the best our knowledge, this is the first study to examine intrarater reliability of the IMS. The Turkish version of the IMS has showed excellent intrarater reliability in our study. These results has demonstrated the IMS has excellent interrater (agreement between different researchers) and intrarater reliability (reproducibility of a practical evaluation), in addition to the other psychometric properties [21]. 

The present study showed that the Turkish version of the IMS demonstrates a good concurrent construct validity with divergent and convergent validity. Twelve out of 13 predefined hypotheses were confirmed (92%) indicating that the construct validity of the IMS was good. Tipping et al. demonstrated that the IMS has proof of construct validity including convergent (there was a correlation between the IMS and muscle strength, and there was a statistical difference in the IMS score between with and without ICU-acquired weakness) divergent (there is no correlation between the IMS and weight, and there is no significant difference between male and female) validity [6]. In accordance with the literature, the Turkish version of the IMS demonstrates a good concurrent construct validity. 

This study had some limitations. Firstly, although we had enough the minimum required sample size, this study was a single-centre (coronary ICU) study. Therefore, generalizabilty of these results across all patients is reduced. Secondly, there was a short time interval to evaluate intrarater reliability. This short time interval between evaluations may affect intrarater reliability results. 

In conclusion, the present study presents the IMS Turkish version is suitable for use in Turkey. This study suggests that the IMS Turkish version is a reliable and valid scale for assessing functional status and mobility level in ICU patients. 

## Informed consent

The study was approved by the Noninvasive Research Ethics Board of Dokuz Eylül University (no.: 3526-GOA, 2017/21–17) and performed following the ethical standards as laid down in the 1964 Declaration of Helsinki (as revised in Brazil 2013). All the participants gave written informed consent before participation in the study.
